# Altered intrinsic functional brain architecture in patients with functional constipation: a surface-based network study

**DOI:** 10.3389/fnins.2023.1241993

**Published:** 2023-09-21

**Authors:** Xiang Yu, Jingjie Yu, Yuwei Li, Jiying Cong, Chao Wang, Ran Fan, Wanbing Wang, Lige Zhou, Chen Xu, Yiming Li, Yawu Liu

**Affiliations:** ^1^Department of Radiology, Tianjin Union Medical Center, Tianjin, China; ^2^Department of Psychiatry and Psychology, Tianjin Union Medical Center, Tianjin, China; ^3^Department of Colorectal Surgery, Tianjin Union Medical Center, Tianjin, China; ^4^Graduate School of Tianjin Nankai University, Tianjin, China; ^5^Graduate School of Tianjin Medical University, Tianjin, China; ^6^Department of Neurology, University of Eastern Finland, Kuopio, Finland; ^7^Department of Clinical Radiology, Kuopio University Hospital, Kuopio, Finland

**Keywords:** functional constipation, surface-based analysis, resting-state fMRI, graph theoretical analysis, functional connectivity

## Abstract

**Background:**

Functional constipation (FCon) is a common functional gastrointestinal disorder (FGID). Studies have indicated a higher likelihood of psychiatric disorders, such as anxiety, depression, sleep disturbances, and impaired concentration, among patients with FCon. However, the underlying pathophysiological mechanisms responsible for these symptoms in FCon patients remain to be fully elucidated. The human brain is a complex network architecture with several fundamental organizational properties. Neurological interactions between gut symptoms and psychiatric issues may be closely associated with these complex networks.

**Methods:**

In the present study, a total of 35 patients with FCon and 40 healthy controls (HC) were recruited for a series of clinical examinations and resting-state functional magnetic imaging (RS-fMRI). We employed the surface-based analysis (SBA) approach, utilizing the Schaefer cortical parcellation template and Tikhonov regularization. Graph theoretical analysis (GTA) and functional connectivity (FC) analysis of RS-fMRI were conducted to investigate the aberrant network alterations between the two groups. Additionally, correlation analyses were performed between the network indices and clinical variables in patients with FCon.

**Results:**

At the global level, we found altered topological properties and networks in patients with FCon, mainly including the significantly increased clustering coefficient (C_*P*_), local efficiency (E_*loc*_), and shortest path length (L_*P*_), whereas the decreased global efficiency (E_*glob*_) compared to HC. At the regional level, patients with FCon exhibited increased nodal efficiency in the frontoparietal network (FPN). Furthermore, FC analysis demonstrated several functional alterations within and between the Yeo 7 networks, particularly including visual network (VN), limbic network (LN), default mode network (DMN), and somatosensory-motor network (SMN) in sub-network and large-scale network analysis. Correlation analysis revealed that there were no significant associations between the network metrics and clinical variables in the present study.

**Conclusion:**

These results highlight the altered topological architecture of functional brain networks associated with visual perception abilities, emotion regulation, sensorimotor processing, and attentional control, which may contribute to effectively targeted treatment modalities for patients with FCon.

## Introduction

Functional constipation (FCon) is a common functional gastrointestinal disorder (FGID) affecting approximately 15% of the global population (Bharucha and Lacy, 2020). It is defined by the Rome IV criteria, which include infrequent bowel movement, painful defecation, excessive straining, hard and/or large stools, and a sensation of incomplete evacuation (Rao et al., 2016; Vriesman et al., 2020; Sharma et al., 2021). The severity of these symptoms can vary among patients and tends to increase with age. FCon has a significant impact on an individual’s quality of life and imposes a considerable healthcare burden. Like many other FGID, it seems that FCon is more prevalent in female, with an average female-male ratio of 2.1:1 (Mugie et al., 2011). Studies have indicated a higher likelihood of psychiatric disorders, such as anxiety, depression, sleep disturbances, and impaired concentration, among patients with FCon (Vriesman et al., 2020; Person and Keefer, 2021). However, the underlying pathophysiological mechanisms responsible for these symptoms in FCon patients remain to be fully elucidated. The gut-brain connection is known to affect human emotion, stress, motivation, and higher cognitive function, and maintaining homeostasis of the gastrointestinal tract (Mayer et al., 2019). The hypothesis of gut-brain axis dysfunction may be the underlying mechanism for the comorbidity of FCon and psychiatric factors, with complex interactions and bidirectional communication between the central nervous system (CNS) and enteric nervous system (ENS) (Jang et al., 2020).

With the advent of non-invasive techniques for mapping brain function in humans, functional brain imaging studies in FGID have provided valuable insights into the spontaneous and evoked brain features and the role of gut-brain interaction in both health and disease (Mayer et al., 2019). Specifically, neuroimaging studies conducted on patients with FCon have demonstrated structural and functional abnormalities in various brain regions, highlighting their involvement in emotion processing modulation, somatic and sensory processing, and motor control (Zhu et al., 2016; Hu et al., 2020). The human brain is a complex network architecture with several fundamental organizational properties (Fox, 2018). Neurological interactions between gut symptoms and psychiatric issues may be closely associated with these complex networks. In our previous study, we conducted graph theoretical analysis (GTA) and functional connectivity (FC) analysis using resting-state functional magnetic resonance imaging (RS-fMRI) to investigate intrinsic brain functional networks and identified significant differences between individuals with FCon and healthy controls (HC). Compared to the HC, patients exhibited a considerable increase in assortativity at the global level. There was a significant increase in nodal degree and efficiency in the sensorimotor network (SMN), coupled with a reduction in nodal degree but an increase in efficiency within the default mode network (DMN) at the regional level. Additionally, the sub-network and large-scale analysis displayed alterations in various functional brain networks particularly within and between the visual network (VN) and SMN (Yu et al., 2023). Similarly, Liu et al. (2018) investigated functional brain topological organization in 42 patients with FCon and 41 HC. Their findings indicated that the thalamo-cortical network, comprising the thalamus, rostral anterior cingulate cortex (rACC), and somatosensory-motor area, exhibited significantly lower normalized clustering coefficient and small-worldness. Furthermore, they observed abnormal interactions with the limbic network (Liu et al., 2021). Recent studies reported significantly aberrant changes in brain network in patients with FCon using independent component analysis (ICA). Zhang D. et al. (2022) investigated the large-scale interaction of RS-fMRI brain networks in 20 FCon patients and found significant differences within the basal ganglia network (BGN), DMN, and between resting-state networks (RSNs), particularly involving connections to VN. A recent publication investigated the differences in RSNs and further analyzed the alterations in FC between RSNs in a cohort of 39 female patients with FCon and 36 female HC. The study found decreased FC in the salience network (SN)-right control executive network (CEN), increased FC in the SN-BGN and DMN-left CEN with significant difference of pairwise FC strengths (Zhang L. et al., 2022).

Patterns of brain abnormalities in FCon have not been consistently reproducible due to the different methodological approaches used in previous studies. Moreover, these studies primarily relied on traditional volume-based analysis, which presented a major problem of imprecise cortical positioning (Oosterhof et al., 2011; Zuo et al., 2013). It is important to note that the function of the brain exhibits a surface-based organization (Yan et al., 2021). The traditional volume-based approach was reported to achieve only 35% of the precision of the best surface-based analysis (SBA) (Coalson et al., 2018). In contrast, SBA can provide several advantages over traditional volume-based methods. Firstly, the SBA is particularly advantageous for analyzing brain regions with complex curved structures, such as the occipital lobe or orbitofrontal cortex (Wang et al., 2015; Rudebeck and Rich, 2018). By mapping fMRI data directly onto the curved cortical surface, it preserves the anatomical integrity of these regions and allows for more accurate characterization of functional activity within them, compared to volume-based methods that may suffer from spatial distortions due to anatomical variability. Secondly, the fMRI signal is often contaminated by signals from non-brain tissues (e.g., cerebrospinal fluid, skull) in volume-based analysis. The SBA restricts the analysis to the cortical surface, minimizing the influence of non-brain tissues and reducing partial volume effects. This results in a cleaner and more specific representation of cortical activity. Furthermore, the surface-based representation of fMRI data provides a common anatomical framework that can facilitate direct comparisons of brain activity across different individuals or studies. This allows for more reliable and meaningful group-level analyses, enhancing the reproducibility and generalizability of findings. Overall, the surface-based method in fMRI offers efficient analysis of curved brain structures, improved spatial specificity, anatomical accuracy, reduction of partial volume effects, and facilitation of cross-subject comparisons, making it a valuable neuroimaging method (Oosterhof et al., 2011; Coalson et al., 2018). Additionally, the use of high-resolution templates in the analysis can provide more detailed information about network alterations (Wang et al., 2009). In order to enhance the functionally and connectionally homogeneous of cerebral cortex parcellation, a surface-based Schaefer400 cortical template was applied in this study, which based on gradient-weighted Markov Random Field (gwMRF) model (Schaefer et al., 2018). To date, there is no widely accepted standard for estimating FC. However, the Tikhonov regularization, also known as L2-norm ridge regression, has been shown it provides higher predictive power and more stable inversion for the covariance matrix compared to using full correlation/covariance and unregularized partial correlation (Pervaiz et al., 2020). In the present study, we adopted a SBA approach using a high-resolution template and Tikhonov regularization. This allowed us to employ GTA and FC analysis of RS-fMRI to investigate aberrant network alterations in the brain among patients with FCon. We focused on exploring the aberrant brain network features in disorders of gut-brain interaction in FCon and hypothesized that within and between intrinsic brain networks may show significant alterations, and the changes may correlate with the clinical variables, which may provide deeper insight into improved understanding of the underlying pathophysiological mechanism associated with gut dysfunction at the surface-based level and contribute to the development of more effectively targeted treatment modalities for patients with FCon.

## Materials and methods

### Participants

Patients with FCon were recruited from the Tianjin Union Medical Center, while healthy volunteers were recruited from the local community. FCon diagnosis was established by a gastroenterologist who had experience in diagnosing FGID using the Rome IV criteria. In order to exclude patients who met the criteria for irritable bowel syndrome (IBS), individuals with FCon did not experience abdominal pain as a predominant symptom or it occurred less than once per week (Drossman and Hasler, 2016). Patients who were using opiates were excluded from a diagnosis of FCon, as their symptoms were more indicative of opioid-induced constipation. All patients underwent colonoscopy to rule out any secondary conditions such as colon cancer and inflammatory bowel disease. High resolution anorectal manometry was employed to evacuate suspected rectal evacuation disorder. Moreover, a radio-opaque marker test was used to assess colonic transit. Patients with conditions such as redundant sigmoid colon, congenital giant colon, pelvic floor muscle relaxation, constipation after childbirth, and neurological/mental/medical disorders requiring immediate treatment were excluded. Individuals who smoked cigarettes, abused alcohol, or were taking medications that could impact the central nervous system were also excluded. All participants, both patients and HC, were provided with a series of self-administered questionnaires to complete. These included the Mini-Mental State Examination (MMSE), Zung Self-rating Depressive Status Scale (SDS), Zung Self-rating Anxiety Scale (SAS), and Wexner constipation scoring system questionnaires (Zung, 1965, 1971; Folstein et al., 1975; Agachan et al., 1996).

### MRI data acquisition

Brain MR imaging was performed using a 3.0 T Siemens Magnetom Skyra scanner. During the resting state scanning, participants were instructed to remain still in the scanner with their eyes closed. They were asked not to think about anything in particular or fall asleep. Although the eyes-closed condition may lead to drowsiness and the possibility of falling asleep, none of the subjects included in this study reported falling asleep during the scanning session. The resting state scan lasted for 6 min and 19 s, during which 180 functional images were collected. Functional images were acquired using a T2-weighted gradient-echo echo-planar imaging (EPI) sequence with the following parameters: flip angle 90, repetition time (TR) 2000 ms, echo time (TE) 30 ms, matrix 72 × 72, field of view (FOV) 220 mm × 220 mm, slice thickness of 3.0 mm with a 1.0 mm gap, 36 interleaved axial slices covering the entire brain. Following the resting-state scan, structural images were acquired for each participant to facilitate spatial normalization. Structural images were acquired using a 3D T1-weighted magnetization-prepared rapidly acquired gradient-echo (MPRAGE) sequence with the following parameters: flip angle 8°, TR 2000 ms, TE 2.34 ms, matrix 256 × 256, FOV 256 mm × 256 mm, spatial resolution 1 mm× 1 mm× 1 mm, thickness 1 mm, and 192 sagittal slices.

### Image preprocessing

The SBA was performed using the toolbox for Data Processing and Analysis for Brain Imaging on Surface (DPABISurf_V1.7) (Yan et al., 2021) within the fMRIPrep 20.2.5, which is based on Nipype 1.6.1. For more details about the pipeline, please refer to the workflows section in the fMRIPrep’s documentation (Esteban et al., 2019). The data preprocessing pipeline in the present study consisted of the following steps: (1) fMRI data distortions caused by inhomogeneity in the main (B0) magnetic field by utilizing a field map for correction. (2) Exclusion of the initial 10 functional images to allow for signal stabilization. (3) Conversion of the data into the Brain Imaging Data Structure (BIDS) format. (4) Preprocessing of anatomical data, including the following procedures: The T1-weighted (T1w) image was corrected for intensity non-uniformity (INU) with N4BiasFieldCorrection, distributed with ANTs 2.3.3, and used as T1w-reference throughout the workflow. The T1w-reference was then skull-stripped with a Nipype implementation of the antsBrainExtraction.sh workflow (from ANTs), using OASIS30ANTs as target template. Brain tissue segmentation of cerebrospinal fluid (CSF), white-matter (WM) and gray-matter (GM) was performed on the brain-extracted T1w using fast (FSL 5.0.9). Brain surfaces were reconstructed using recon-all (FreeSurfer 6.0.1). (5) Functional data preprocessing as follows: First, a reference volume and its skull-stripped version were generated using a custom methodology of fMRIPrep. The blood oxygen level dependent (BOLD) reference was then co-registered to the T1w reference using bbregister (FreeSurfer) which implements boundary-based registration. BOLD runs were slice-time corrected using 3dTshift. The BOLD time series were resampled to surfaces on the fsaverage5 space. (6) Then, nuisance signals were regressed out, including white matter signal, cerebrospinal fluid signal, linear trend, global mean signal and the signal associated with the 24 Friston head-motion parameters. (7) Finally, the normalized functional images were smoothed with a 6-mm full-width at half-maximum (FWHM) kernel and bandpass filtered (0.01–0.1 Hz). Participants with a maximum head motion larger than 3 mm in displacement or 3° in rotation were excluded from further analysis, as well as those with mean frame-wise displacement (FD) larger than 0.2 mm. In total, ten patients and five HC were excluded in the current study due to these motion criteria. Considering the possible confounding effect of micromovements on resting state BOLD signals, mean FD values were calculated for each participant and compared between the two groups using the Jenkinson formula, which could reflect the temporal derivative of the movement parameters (Jenkinson et al., 2002).

### Network construction

A functional network was constructed using two fundamental elements: nodes and edges. Nodes of the functional brain network represent brain regions, and network edges represent interregional resting-state FC. To define the nodes, we utilized the Schaefer atlas, which divides the brain into 400 areas distributed throughout. Each node was represented as a sphere with a radius of 1 mm. Mean time series were extracted from all vertices within each node and then averaged. To quantify the Tikhonov regularization-based correlation, we employed the DPABINet_V1.2 toolbox, which is a module within DPABI (Yan et al., 2016). For each node, the edge-based FC was computed between any pair of two nodes using BOLD signals. These FC values were transformed into z-scores using Fisher’s r-to-z formula, which improves normality. As a result, we obtained a symmetrical 400 × 400 matrix of Z-values for each subject. To investigate the network’s topological properties, we calculated binary topological parameters of the FC matrices across a wide range of network edge sparsities. In the present study, the sparsity threshold ranged from 10 to 34% with an interval of 1%.

### Network analysis

Based on the constructed brain networks, we analyzed both global and regional topological metrics of brain graphs. At the global level, we investigated several network metrics, including the clustering coefficient (C_*P*_), shortest path length (L_*P*_), and their normalized versions using a random network (γ, λ). We also calculated global efficiency (E_*glob*_), local efficiency (E_*loc*_), and assortativity. At the regional level, we examined nodal degree, betweenness, and nodal efficiency for each individual node in the network. For each metric, the area under the curve (AUC) for this density range was calculated as representative to avoid any bias introduced by focusing on specific density values. The significant nodes were classified and reported based on the networks defined by Yeo et al. (2011), including the visual network (VN), somatosensory-motor network (SMN), dorsal attention network (DAN), ventral attention network (VAN), limbic network (LN), frontoparietal network (FPN), and default mode network (DMN).

### Statistical analysis

Demographic and clinical variables were analyzed by using SPSS Statistics (Version 26, IBM) software. Kolmogorov-Smirnov normality test was used to identify the distribution mode of clinical information, questionnaires’ scores, motion parameters, and the metrics of global and regional networks. Two sample *t*-test was applied to examine the differences in most of clinical information and scale scores between FCon and HC groups. The chi-square test was used to compare differences in sex distribution. The Mann–Whitney *U* test was used to analyze the duration of illness, MMSE, and mean values of FD, respectively.

To test the difference in AUC of each graph theoretical network metric at both global and regional levels between the two groups, we performed permutation tests with Permutation Analysis of Linear Models (PALM) (Winkler et al., 2016). For the between-group contrasts, age, sex, mean values of FD, and cortex volume were added in the linear model as covariates. The permuted data was utilized to record the maximum statistic, thereby constructing a null distribution. This procedure was repeated 10,000 times in order to compare the null distribution of maximum statistics with the observed statistics, enabling estimation of a *P*_*perm*_ value for each attribute. The utilization of False Discovery Rate (FDR) correction for multiple comparison correction with a significance threshold of *P* < 0.05.

Furthermore, the Network Based Statistic (NBS) was used to assess detailed differences in sub-network FC between FCon and HC participants while using age, sex, mean values of FD, and cortex volume as covariates. The NBS analysis is a validated, non-parametric statistical approach for controlling family wise error in connectome analysis (Zalesky et al., 2010). A sub-network was defined by interconnectedness of suprathreshold edges in topological space. The primary threshold was set at *P* < 0.001 (two tailed) for every edge. Next, the size of the connected components of this thresholded network was computed. In this context, every connected component was sets of nodes that can be linked by a set of suprathreshold connections. The statistical significance of the size of each observed component was evaluated by comparing them to an empirical null distribution generated by randomly permuting the group membership of each individual (10,000 permutations). The corrected *P*-value for each observed component was estimated based on the proportion of null component sizes larger than the observed size. Two directions of the contrasts in NBS analysis were tested separately, and one-tailed *P* value was derived so that components with *P* < 0.025 was taken as significant sub-networks differentiating the two groups.

We further analyzed the FC of large-scale within- and between-network connectivity by calculating the average FC z-scores across all relevant edges. In our analysis, we did not treat the negative associations as absolute values; therefore, both negative and positive correlations were averaged together in the large-scale network analysis. With the definition of seven networks, this resulted in seven averaged FC values within the networks and twenty-one averaged FC values between the networks. To compare group differences of FC in the large-scale network, we conducted permutation tests while taking age, sex, mean values of FD, and cortex volume as covariates. This step was repeated 10,000 times. To account for multiple comparisons, we used the FDR correction with a significance threshold of *P* < 0.05, two-tailed.

Finally, Pearson’s correlation analysis between the network indices and clinical variables in patients was performed after controlling for the influences of age, sex, mean values of FD, and cortex volume. We applied FDR correction for multiple comparisons, and the statistical significance was set to *P* < 0.05.

## Results

### Demographic characteristics and motion parameters

As shown in Table 1, thirty-five right-handed patients (age 47.2 ± 12.8 years, 6 males) and forty age- and sex- matched right-handed HC (age 45.1 ± 13.1 years, 13 males) completed the MRI scans. There were significant differences in the SAS (*P* < 0.0001), and SDS (*P* < 0.0001), but no significant differences in age, sex, education level, BMI, MMSE, and mean values of FD between the two groups (*P* > 0.05).

**TABLE 1:** Demographic, clinical information, and motion parameters of participants.

Characteristics	FCon (*N* = 35)	HC (*N* = 40)	*P*-value
Age (years)	47.2000 (12.7552)	45.0500 (13.1460)	0.4760
Sex (M/F)	6/29	13/27	0.127
Education level (years)	12.3429 (2.8691)	14.1000 (4.9500)	0.0608
BMI (Kg/M^2^)	22.8888 (2.4822)	22.6846 (3.0218)	0.7521
Duration of illness (years)	10 (4,20)	NA	NA
MMSE	30 (30,30)	30 (29.25,30)	0.1983
SAS	49.8000 (7.2266)	35.0563 (8.9944)	<0.0001*
SDS	53.1143 (8.9501)	36.8813 (12.0722)	<0.0001*
Wexner constipation score	13.4857 (4.3343)	NA	NA
Mean values of FD	0.0552 (0.0463, 0.0828)	0.0507 (0.0402, 0.0733)	0.303

Values shown are mean (count number for sex), statistics, and *P*-value of two-sample *t*-tests comparing patients with FCon and HC (Chi-square test for sex and Mann–Whitney *U* test for duration of illness, MMSE, and Mean values of FD, respectively).

Standard deviations or quartile are shown in parentheses.

FCon, functional constipation; HC, healthy controls; M, male; F, female; BMI, body mass index; MMSE, Mini-mental State Examination; SAS, ZUNG Self-rating Anxiety Scale; SDS, ZUNG Self-rating Depressive Scale.

* *P* < 0.05.

### Global topological networks

**FIGURE 1:**
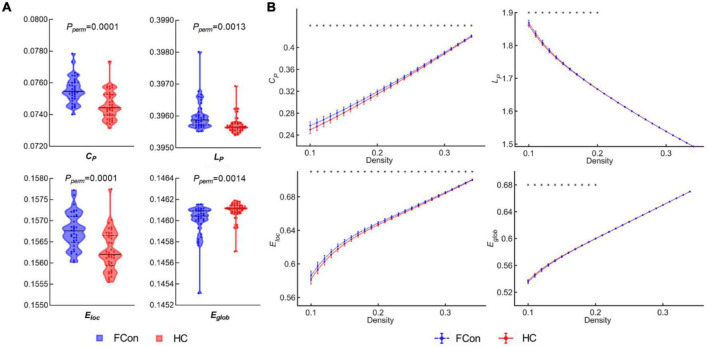
Between-group differences in global network topological metrics. **(A)** Violin plots show the area under the curve (AUC) parameters of the clustering coefficient (C_*P*_), shortest path length (L_*P*_), local efficiency (E_*loc*_) and global efficiency (E_*glob*_) for patients with FCon and HC. **(B)** C_*P*_, L_*P*_, E_*loc*_, and E_*glob*_ across a wide range of density thresholds between 10 and 34%. Each point and error bar denote the mean and standard deviation at each density level, respectively. * indicates a significant difference at a given density threshold (*P* < 0.05, FDR correction).

For the AUC across density range of 10 to 34%, compared with the HC, we found significantly increased C_*P*_, E_*loc*_ and L_*P*_ (Figure 1), whereas, significantly decreased E_*glob*_ in patients with FCon (*P* < 0.05, FDR correction). There were no significant group differences in assortativity, γ, λ, and σ after FDR correction.

### Regional nodal characteristics

**FIGURE 2:**
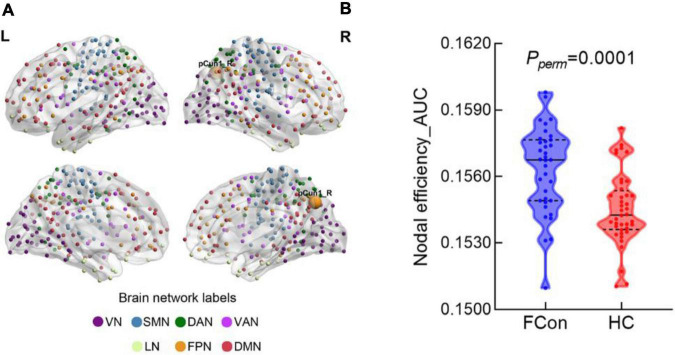
Between-group difference in nodal efficiency at the regional level. **(A)** There was significant difference in the pCun1_R which reported based on Yeo 7 networks. **(B)** Violin plot shows the AUC metric of the brain region with significant difference in nodal efficiency for patients with FCon and HC (*P* < 0.05 after FDR correction). VN, visual network; SMN, somatosensory network; DAN, dorsal attention network; VAN, ventral attention network; LN, limbic network; FPN, frontoparietal network; DMN, default mode network.

Compared with the HC, the patients with FCon showed increased nodal efficiency (AUC) in the FPN (e.g., right Cont-pCun1) (Figure 2 and Table 2). There were no significant group effects on nodal degree and betweenness (AUC) after FDR correction.

**TABLE 2:** Group differences in topological metrics between FCon and HC in regional level.

Metrics (AUC) Nodal efficiency FCon > HC	ROI	fsaverage5 coordinates			FCon (*n* = 35) Mean ± SD	HC (*n* = 40) Mean ± SD	*P*-value
		*x*	*y*	*z*			
	pCun1_R	12	−69	38	0.1563 ± 0.0019	0.1545 ± 0.0016	0.0001

AUC, area under the curve; SD, standard deviations.

*P* < 0.05 (FDR corrected).

For each ROIs abbreviation, please refer to the Supplementary Table 1.

### Sub-network connectivity

**FIGURE 3:**
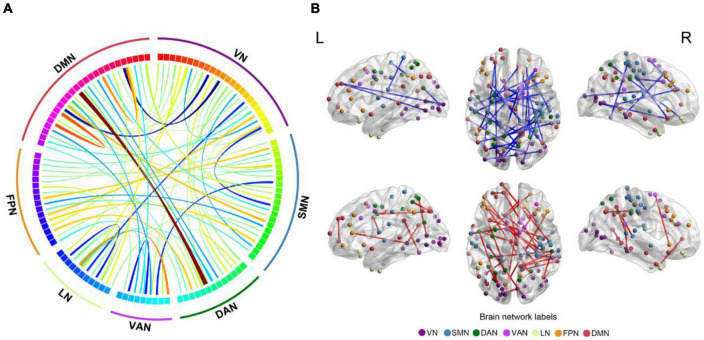
Differences in FC between FCon and HC in sub-network analysis. **(A,B)** The 119 nodes defined by Schaefer 400-area cortical parcellation atlas together with significant 72 unique Yeo 7 network-pair edges in patients with FCon are list in circos and brain map, respectively. For the color of edges, cool color indicates the FC is decreased while warm color indicates the FC is increased (*P* < 0.001 after NBS correction). For network abbreviation, please refer to the legend of Figure 2.

**FIGURE 4:**
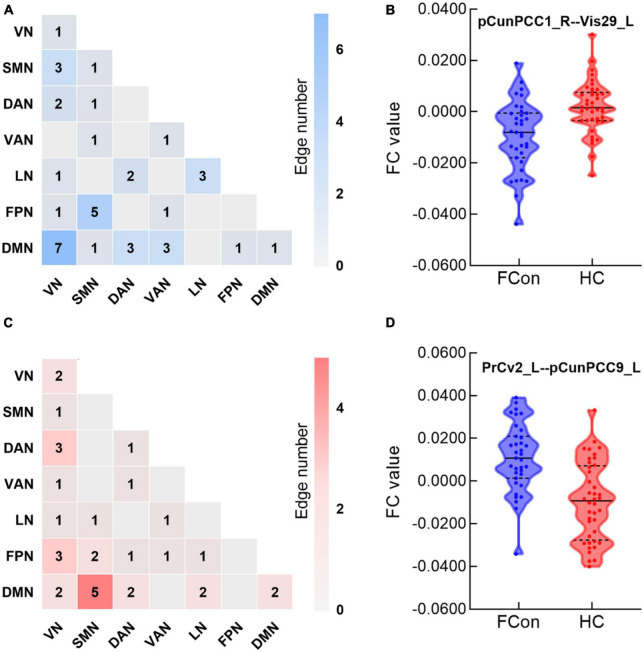
Between-group differences in edge-based FC. **(A,C)** Heatmaps show the number of significant edges for each pair of networks in group differences. Blue indicates that the FC is decreased while red indicates that the FC is increased compared with HC. **(B,D)** Violin plots show edges corresponding to the most significant difference in the decreased or increased FC for FCon and HC, respectively (*P* < 0.001 after NBS correction). For network abbreviation, please refer to the legend of Figure 2.

In total, the NBS analysis identified significant clusters (*P* < 0.001) consisting of 119 nodes and 72 edges (hypo/hyper edges = 39/33) (Figures 3, 4 and Table 3). The suprathreshold edges with decreased and increased FC involved all of Yeo 7 networks. Specifically, more affected edges were linked with the nodes within the VN, LN, and DMN, and between-networks, including the VN-DMN, SMN-FPN, and SMN-DMN (Supplementary Table 2). In addition, the FC was most significantly decreased in the edge of Vis29_L–Default-pCunPCC1_R. The FC was most significantly increased in the edge of DorsAttn-PrCv2_L-Default-pCunPCC9_L (Figure 4).

**TABLE 3:** Number and ratio of pair-wise FC difference between FCon and HC.

Networks	VN		SMN		DAN		VAN		LN		FPN		DMN	
	**Hypo**	**Hyper**	**Hypo**	**Hyper**	**Hypo**	**Hyper**	**Hypo**	**Hyper**	**Hypo**	**Hyper**	**Hypo**	**Hyper**	**Hypo**	**Hyper**
VN	1 (0.0546%)	2 (0.1093%)												
SMN	3 (0.0639%)	1 (0.0213%)	1 (0.0342%)	0 (0%)										
DAN	2 (0.0713%)	3 (0.1069%)	1 (0.0282%)	0 (0%)	0 (0%)	1 (0.0966%)								
VAN	0 (0%)	1 (0.0349%)	1 (0.0276%)	0 (0%)	0 (0%)	1 (0.0463%)	1 (0.0925%)	0 (0%)						
LN	1 (0.0631%)	1 (0.0631%)	0 (0%)	1 (0.0500%)	2 (0.1672%)	0 (0%)	0 (0%)	1 (0.0818%)	3 (0.9231%)	0 (0%)				
FPN	1 (0.0315%)	3 (0.0946%)	5 (0.1249%)	2 (0.0500%)	0 (0%)	1 (0.0418%)	1 (0.0409%)	1 (0.0409%)	0 (0%)	1 (0.0740%)	0 (0%)	0 (0%)		
DMN	7 (0.1261%)	2 (0.0360%)	1 (0.0143%)	5 (0.0714%)	3 (0.0717%)	2 (0.0478%)	3 (0.0701%)	0 (0%)	0 (0%)	2 (0.0845%)	1 (0.0211%)	0 (0%)	1 (0.0244%)	2 (0.0488%)

Values in each cell are the count absolute number of suprathreshold edges associated with every pair of networks within the significant cluster acquired from the NBS analysis. In contrast, values in the parentheses indicate the percentage ratio of these numbers to the total number of connections for each network pair. Hypo cells represent hypoconnectivity (FCon < HC), whereas hyper cells represent hyperconnectivity (FCon > HC).

### Large-scale network connectivity

**FIGURE 5:**
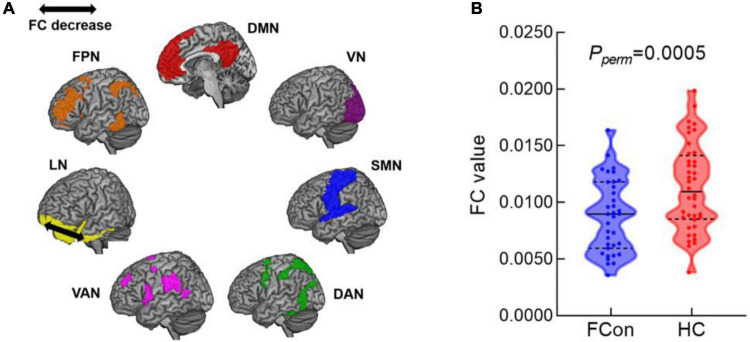
Differences in large-scale network between FCon and HC. **(A)** The schematic diagram shows the network connection with significant FC decrease within LN-LN. **(B)** Violin plot shows FC value of the significant difference within-network for FCon and HC (FDR-corrected *P* < 0.05). For network abbreviation, please refer to the legend of Figure 2.

Compared with the HC, the patients demonstrated decreased within-network FC for one pair of networks: LN-LN after FDR correction (Figure 5). However, the between-network FC values did not significantly differ between the two groups after FDR correction.

### Clinical associations

In the correlation analysis within the FCon group, controlling for age, sex, mean values of FD, and cortex volume. It is worth noting that no significant correlations were found between the network metrics and the SAS, SDS, as well as Wexner constipation score after FDR correction.

### Validation analyses

We employed a different high-resolution cortical atlas and rerun the network analysis. Using the Human Connectome Project Multi-Modal Parcellation (HCP-MMP) atlas (360 areas) (Glasser et al., 2016), permutation tests exhibited significant group changes. Compared with the HC, we found significantly increased C_*P*_, E_*loc*_, and L_*P*_, whereas, significantly decreased E_*glob*_ in patients with FCon (Supplementary Figure 1). The patients with FCon showed increased nodal efficiency in the VN (Supplementary Figure 2 and Supplementary Table 3). Furthermore, for 64,800 pairs of edges (360 × 360/2), FC was computed for each group, and NBS analysis was conducted to examine differences in sub-network FC between the patients and HC participants. The result revealed a significant cluster (*P* < 0.001) consisting of 101 nodes and 60 edges (hypo/hyper edges = 29/31) involved several networks in the FCon group (Supplementary Figure 3 and Supplementary Table 4). In addition, in the large-scale network analysis, we found that compared with the HC, the patients demonstrated significantly decreased FC within network in the LN-LN and between-networks in the SMN-FPN and DMN-VAN after FDR correction (Supplementary Figure 4). These findings were generally similar to those results calculated by using Schaefer atlas, suggesting a satisfied reproducibility in the present study.

## Discussion

The current RS-fMRI study investigated the alterations in intrinsic brain functional networks in patients with FCon using GTA and FC analysis at the surface-based level. The demographic information indicated that patients with FCon experienced more severe emotional problems compared to the HC group. The findings of the study were generally in line with the initial hypothesis and revealed significant alterations in topological properties and networks at both the global and regional levels in FCon patients. At the global level, the patients exhibited increased C_*P*_, shortest L_*P*_, and E_*loc*_, while decreased E_*glob*_ compared to HC. At the regional level, increased nodal efficiency was observed in the FPN of FCon patients. Further analysis using NBS revealed alterations in several functional brain networks, indicating disrupted connectivity patterns and more affected edges connected to nodes, particularly within the VN, LN, and DMN, as well as between networks, including VN-DMN, SMN-FPN, and SMN-DMN. In addition, FC of large-scale network was significantly decreased in one pair of within-networks, including LN-LN. However, correlation analysis revealed that there were no significant associations between the network metrics and clinical variables.

### Altered small-world properties of the surface-based brain network in patients with FCon

Functional brain networks are characterized by specific topological features, with C_*P*_ and L_*P*_ being crucial components for assessing small-worldness network parameters (Mheich et al., 2020). Regular networks typically exhibit high C_*P*_ and long L_*P*_, while random networks tend to demonstrate low C_*P*_ and short L_*P*_. In contrast, a small-world network presents a combination of high C_*P*_ and short L_*P*_, which enables efficient communication and information processing through the integration and segregation of information. In the current study, patients with FCon showed increased C_*P*_ and L_*P*_, indicating increased local efficiency, reduced global efficiency, and a shift toward regular network. The exact underlying mechanism for this regularization process is still uncertain. However, we hypothesized that the increase in C_*P*_ and L_*P*_ may be associated with a decrease in signal transmission speed and coordination. To compensate for this, there is a corresponding increase in local efficiency to maintain effective communication within the network. Further analysis of regional network indices revealed significantly increased nodal efficiency pattern in FPN of patients with FCon. The FPN is involved in goal-directed attention and cognitive control, and responsible for maintaining attention, working memory, and task switching, which highly integrated with other brain networks (Marek and Dosenbach, 2018; Kuchenhoff et al., 2021). It also plays a vital role in decision-making, response selection, and response inhibition providing a functional backbone for rapid and flexible modulation of other brain networks. The increased nodal efficiency in the FPN observed in this study may indicate enhanced control abilities in response to bowel stimulation in patients with constipation.

### Gut-brain axis related surface-based brain network alterations

Abnormal gut-brain connection can lead not only to decrease intestinal movement but also to affect the brain function, especially emotion related dysfunction in patients with FCon, suggesting the potential for personalized treatments that integrate constipation management with targeted antipsychotic medications, rather than only focusing on alleviating constipation symptoms. In the present study, compared with the HC, the patients with FCon experienced constant intestinal symptom together with negative emotion. Meanwhile, both sub-network and large-scale network analysis demonstrated widespread aberrant alterations across the Yeo 7 networks, suggesting the broad impact of FCon on intrinsic functional brain networks at the surface-based level. Specifically, the VN, LN, DMN, and SMN were prominently involved.

The VN is responsible for processing and interpreting visual information. This network enables complex interactions between different parts of the brain, allowing us to process and understand various visual perception (Lu et al., 2020; Wang et al., 2020; Keane et al., 2021). Generally, traditional volume-based neuroimaging studies on patients with FCon showed brain structural and functional abnormalities in several brain regions involved in emotion processing modulation, somatic and sensory processing, and motor control (Hu et al., 2020; Duan et al., 2021; Li G. et al., 2021; Jia et al., 2022). In the present study, most of significant edges are belonging to VN, suggesting its involvement in the pathophysiology of FCon as a key network at the surface-based level. In addition, compared to the volume-based atlas, the surface-based atlas demonstrated a relatively superior ability to preserve the spatial topological structure across the visual system and align with topographic areas (Wang et al., 2009). Surface-based methods may be particularly useful in brain regions with complex curved structures, such as the occipital lobe or orbitofrontal cortex. While previous researches have focused on subcortical regions and the limbic system instead of the visual cortex to study the gut-brain axis (Fan et al., 2019; Liu et al., 2021). Recent studies suggested significantly aberrant alteration of brain networks involving VN in patients with FCon (Zhang D. et al., 2022; Yu et al., 2023). Based on previous studies, the VN is associated with the identification of potentially meaningful visual stimuli for the body such as pain (Lu et al., 2020; Wang et al., 2020; Keane et al., 2021). Additionally, visual distraction has been shown to enhance pain tolerance by diverting one’s attention away from pain, thus reducing its sensory inputs (Gu and Han, 2007; Harvie et al., 2015). Moreover, visual stimuli, like such as viewing pleasant pictures or viewing one’s own hand has also been shown to modulate pain perception (Godinho et al., 2008; Longo et al., 2009). In light of these studies, one approach to establishing a potential link between visual stimuli and functional constipation involves conducting a series of experiments or observational studies. These further studies could entail exposing individuals to various visual stimuli and closely monitoring the subsequent effects on their bowel movements and digestive processes. For example, researchers could present participants with images relating to food and measure and analyze their physiological responses, such as alterations in gut motility and digestion. Adopting this approach can contribute to a more comprehensive comprehension of the potential mechanisms by which visual stimuli influence gastrointestinal function and potentially contribute to the development or worsening of functional constipation.

According to the gut-brain axis hypothesis, the limbic system is a critical component of the gut-brain axis (Mayer et al., 2019). It is currently accepted that prefrontal cortex, amygdala, anterior cingulate cortex, hippocampus, and insula are responsible for emotion and behavior, as well as reward valuation and punishment (Rolls, 2015). In the present study, many altered FC of nodes in NBS-based findings are belong to limbic system, such as orbitofrontal cortex, middle cingulate cortex, and temporal pole. In addition, FC of large-scale network was significantly decreased within LN in patients with FCon. These findings are consistent with previous studies (Jin et al., 2019; Liu et al., 2021). Additionally, the DMN is the network of the brain that processes internal mental states and its activity is often anticorrelated with other intrinsic networks involved in attending to functions such as attentional vigilance and spatial orientation (Yeshurun et al., 2021). Attention control is an important part in the model of emotion that captures emotion dynamics and process model of emotion regulation. Activation of the DMN has been observed in patients with gut diseases (Ma et al., 2015; Weng et al., 2017; Liu et al., 2018; Li L. et al., 2021). Our study revealed that emotional function was impaired in patients with FCon. We could speculate that the altered DMN may affect the anxiety/depression status of patients. The SMN, as a part of the primate cerebral cortex, receives sensory inputs from the periphery, including visceral sensory stimuli, and plays a role in regulating motor function (Kim et al., 2019). Previous researches have demonstrated SMN engagement in FGID and inflammatory bowel disease (Weng et al., 2017; Liu et al., 2018). In the present study, the aberrant FC between-networks including the SMN-FPN, SMN-DMN may be indicative of dysfunctional control abilities or emotion regulation in response to bowel stimulation in the brain following movement of bowel and desire to defecate through a cross-modal somatic mechanism.

It is worth noting that no significant associations were found between the network metrics and psychiatric factors, and constipation symptom after FDR correction. It is discrepant with the results of previous studies (Jin et al., 2019; Zhang et al., 2020; Duan et al., 2021; Li G. et al., 2021; Jia et al., 2022; Zhang L. et al., 2022; Yu et al., 2023). There are two possible reasons. Firstly, in our study, high SDS, SAS, and Wexner constipation scale scores in FCon group were not a criterion for inclusion. Meanwhile, these scales involved in this study are self-assessment scales, which represent objective evaluation of the patient’s subjective experience and thus may affect the results of the correlation analysis. Secondly, alterations in brain activity patterns may be associated with various factors. Head motion and brain structure may have an influence on the results. In previous studies, these variables were not treat as covariates for further analysis, whereas we considered these factors as confounding factors in the current study and used them as covariates when computing the functional brain networks parameters.

### Limitations

Firstly, the sample size was relatively small due to strict exclusion criteria. This limited the generalizability of our findings and may have reduced the statistical power of the study. In future research, it is essential to include a larger cohort to improve the robustness and generalizability of the results. Secondly, the surface-based method used in our study focused solely on the gray matter surface, which unfortunately neglected the subcortical gray matter regions that play a crucial role in brain function. Incorporating subcortical analysis in conjunction with volume-based results would provide a more comprehensive understanding of the alterations in FCon. We are committed to continuously improving our research methods to gain deeper insights into the complexities of FCon and its relationship with brain function in future work. Thirdly, it is essential to include additional clinical subtypes of FCon, classified according to depression and anxiety, as well as a category encompassing patients solely with emotional difficulties without FCon. Further investigations should be conducted to explore the correlation between the clinical presentations and changes in brain function across multiple subtypes. Moreover, our study primarily focused on revealing the intrinsic brain network alterations in FCon. However, further investigations are needed to explore the effects of neuro-feedback treatments, such as repetitive transcranial magnetic stimulation (rTMS), in patients with FCon. Implementing intervention designs would offer deeper insights into the underlying neural mechanisms of the disorder and potentially identify treatment biomarkers associated with FCon. Lastly, future studies could consider conducting multi-center fMRI analyses using deep learning approaches. This approach would enhance the reliability, sensitivity, and specificity of the network parameters.

## Conclusion

The present study aimed to investigate the alterations of intrinsic brain networks at the surface-based level in patients with FCon compared to HC. By utilizing GTA, sub-network, and large-scale FC analysis of RS-fMRI data, we examined the disrupted network architecture in patients with FCon. Our results revealed significant alterations in the graph-theoretical networks of FCon patients at both the global and regional levels, as well as aberrant FC within or between several brain networks. Overall, our findings provide insights into the altered topological architecture of surface-based functional brain networks in FCon. These alterations may underlie the visual perception abnormalities, emotional disturbances, disrupted sensorimotor processing, and attentional deficits observed in patients with FCon. Understanding the specific network alterations associated with FCon could contribute to the development of targeted treatment modalities that address the underlying neurobiological mechanisms of the disorder.

## Data availability statement

The raw data supporting the conclusions of this article will be made available by the authors, without undue reservation.

## Ethics statement

This study involving human participants was reviewed and approved by Ethics Committee of Tianjin Union Medical Center (No. 2022-B54). All patients/participants provided their written informed consent to participate in this study.

## Author contributions

XY, YML, YuL, CX, and JY initiated the study. YML conceived the original idea. CX participated in study design and performed the diagnosis of FCon. XY obtained ethics approval and wrote a first draft of the manuscript. YWL participated in manuscript editing. JY participated in evaluation of clinical psychological scales. JC assisted in recruitment of subjects and analysis of data. CW contributed to the acquisition of fMRI data. RF, WW, and LZ contributed to the data analysis and assisted in execution of the study. All authors contributed to the final manuscript.
