# Supplementary material for: Altered intrinsic functional brain architecture in patients with functional constipation: a surface-based network study
Source: Front Neurosci. 2023 Sep 21;17:1241993. doi: 10.3389/fnins.2023.1241993 (PMC10551127; doi:10.3389/fnins.2023.1241993)
Supplement: Supplementary file 1 [file Data_Sheet_1.docx]

**Supporting Information(SI)**

**Materials and Methods**

The principle and details of Tikhonov regularization

The principle of Tikhonov regularization is to add a balance or trade-off between the residual sum of squares (RSS) and the size of the estimated coefficients. It does this by adding a penalty term to the cost function, which is the square of the Euclidean norm of the coefficient vector.

Without regularizations, the ordinary least squares will minimize:

RSS = ∑(y - Xβ)^2

In Tikhonov regularization, the objective becomes:

RSS + λ||β||^2 = ∑(y - Xβ)^2 + λ||β||^2

The term λ||β||^2 is the penalty term where λ (Lambda) is a tuning parameter that is chosen to balance the trade-off, and it is always non-negative. β represents the coefficient.

As the value of λ increases, the penalty for large coefficients also increases. Thus, it tends to create simpler models with smaller coefficients.

**References**

1.Golub, G.H., Hansen, P.C., O’Leary, D.P., 1999. Tikhonov regularization and total least

squares. SIAM J. Matrix Anal. Appl. 21 (1), 185–194. doi:10.1137/S0895479897326432.

2.Pervaiz U, Vidaurre D, Woolrich MW, Smith SM. Optimising network modelling methods for fMRI. NeuroImage. 2020;211:116604. doi:10.1016/j.neuroimage.2020.116604.

3.Li Y, Cheng P, Liang L, et al. Abnormal resting-state functional connectome in methamphetamine-dependent patients and its application in machine-learning-based classification. Front Neurosci. 2022;16:1014539. doi:10.3389/fnins.2022.1014539.

**Supplementary TABLE 1** Regions of interest used in the present study.

| **Index** | **Region** | **Hemisphere** | **Network** |
| --- | --- | --- | --- |
| 3 | Vis3_L | Left | VN |
| 7 | Vis7_L | Left | VN |
| 8 | Vis8_L | Left | VN |
| 9 | Vis9_L | Left | VN |
| 11 | Vis11_L | Left | VN |
| 13 | Vis13_L | Left | VN |
| 14 | Vis14_L | Left | VN |
| 16 | Vis16_L | Left | VN |
| 18 | Vis18_L | Left | VN |
| 19 | Vis19_L | Left | VN |
| 22 | Vis22_L | Left | VN |
| 29 | Vis29_L | Left | VN |
| 30 | Vis30_L | Left | VN |
| 31 | Vis31_L | Left | VN |
| 32 | SomMot1_L | Left | SMN |
| 40 | SomMot9_L | Left | SMN |
| 42 | SomMot11_L | Left | SMN |
| 46 | SomMot15_L | Left | SMN |
| 57 | SomMot26_L | Left | SMN |
| 67 | SomMot36_L | Left | SMN |
| 73 | DorsAttn-Post5_L | Left | DAN |
| 79 | DorsAttn-Post11_L | Left | DAN |
| 80 | DorsAttn-Post12_L | Left | DAN |
| 82 | DorsAttn-Post14_L | Left | DAN |
| 83 | DorsAttn-Post15_L | Left | DAN |
| 87 | DorsAttn-FEF2_L | Left | DAN |
| 88 | DorsAttn-FEF3_L | Left | DAN |
| 91 | DorsAttn-PrCv2_L | Left | DAN |
| 107 | SalVentAttn-Med1_L | Left | VAN |
| 108 | SalVentAttn-Med2_L | Left | VAN |
| 110 | SalVentAttn-Med4_L | Left | VAN |
| 114 | Limbic-OFC1_L | Left | LN |
| 117 | Limbic-OFC4_L | Left | LN |
| 119 | Limbic-TempPole1_L | Left | LN |
| 120 | Limbic-TempPole2_L | Left | LN |
| 121 | Limbic-TempPole3_L | Left | LN |
| 129 | Cont-Par3_L | Left | FPN |
| 132 | Cont-Par6_L | Left | FPN |
| 135 | Cont-PFCl1_L | Left | FPN |
| 136 | Cont-PFCl2_L | Left | FPN |
| 139 | Cont-PFCl5_L | Left | FPN |
| 142 | Cont-PFCl8_L | Left | FPN |
| 143 | Cont-PFCv1_L | Left | FPN |
| 144 | Cont-pCun1_L | Left | FPN |
| 146 | Cont-Cing1_L | Left | FPN |
| 153 | Default-Temp5_L | Left | DMN |
| 157 | Default-Temp9_L | Left | DMN |
| 164 | Default-Par6_L | Left | DMN |
| 165 | Default-Par7_L | Left | DMN |
| 171 | Default-PFC6_L | Left | DMN |
| 176 | Default-PFC11_L | Left | DMN |
| 179 | Default-PFC14_L | Left | DMN |
| 180 | Default-PFC15_L | Left | DMN |
| 186 | Default-PFC21_L | Left | DMN |
| 191 | Default-pCunPCC2_L | Left | DMN |
| 193 | Default-pCunPCC4_L | Left | DMN |
| 198 | Default-pCunPCC9_L | Left | DMN |
| 202 | Vis2_R | Right | VN |
| 203 | Vis3_R | Right | VN |
| 207 | Vis7_R | Right | VN |
| 216 | Vis16_R | Right | VN |
| 217 | Vis17_R | Right | VN |
| 219 | Vis19_R | Right | VN |
| 220 | Vis20_R | Right | VN |
| 222 | Vis22_R | Right | VN |
| 223 | Vis23_R | Right | VN |
| 225 | Vis25_R | Right | VN |
| 231 | SomMot1_R | Right | SMN |
| 235 | SomMot5_R | Right | SMN |
| 236 | SomMot6_R | Right | SMN |
| 237 | SomMot7_R | Right | SMN |
| 238 | SomMot8_R | Right | SMN |
| 239 | SomMot9_R | Right | SMN |
| 249 | SomMot19_R | Right | SMN |
| 251 | SomMot21_R | Right | SMN |
| 252 | SomMot22_R | Right | SMN |
| 255 | SomMot25_R | Right | SMN |
| 256 | SomMot26_R | Right | SMN |
| 257 | SomMot27_R | Right | SMN |
| 259 | SomMot29_R | Right | SMN |
| 264 | SomMot34_R | Right | SMN |
| 270 | SomMot40_R | Right | SMN |
| 276 | DorsAttn-Post6_R | Right | DAN |
| 279 | DorsAttn-Post9_R | Right | DAN |
| 281 | DorsAttn-Post11_R | Right | DAN |
| 285 | DorsAttn-Post15_R | Right | DAN |
| 286 | DorsAttn-Post16_R | Right | DAN |
| 297 | SalVentAttn-TempOccPar4_R | Right | VAN |
| 310 | SalVentAttn-PFCl1_R | Right | VAN |
| 311 | SalVentAttn-Med1_R | Right | VAN |
| 314 | SalVentAttn-Med4_R | Right | VAN |
| 317 | SalVentAttn-Med7_R | Right | VAN |
| 318 | SalVentAttn-Med8_R | Right | VAN |
| 319 | Limbic-OFC1_R | Right | LN |
| 321 | Limbic-OFC3_R | Right | LN |
| 322 | Limbic-OFC4_R | Right | LN |
| 325 | Limbic-TempPole1_R | Right | LN |
| 327 | Limbic-TempPole3_R | Right | LN |
| 329 | Limbic-TempPole5_R | Right | LN |
| 340 | Cont-PFCv1_R | Right | FPN |
| 348 | Cont-PFCl8_R | Right | FPN |
| 349 | Cont-PFCl9_R | Right | FPN |
| 351 | Cont-PFCl11_R | Right | FPN |
| 352 | Cont-PFCl12_R | Right | FPN |
| 357 | Cont-pCun2_R | Right | FPN |
| 360 | Cont-PFCmp1_R | Right | FPN |
| 366 | Default-Par5_R | Right | DMN |
| 367 | Default-Temp1_R | Right | DMN |
| 370 | Default-Temp4_R | Right | DMN |
| 371 | Default-Temp5_R | Right | DMN |
| 373 | Default-Temp7_R | Right | DMN |
| 375 | Default-PFCv1_R | Right | DMN |
| 381 | Default-PFCdPFCm3_R | Right | DMN |
| 390 | Default-PFCdPFCm12_R | Right | DMN |
| 392 | Default-pCunPCC1_R | Right | DMN |
| 397 | Default-pCunPCC6_R | Right | DMN |
| 398 | Default-pCunPCC7_R | Right | DMN |
| 399 | Default-pCunPCC8_R | Right | DMN |
| 400 | Default-pCunPCC9_R | Right | DMN |
| Abbreviations: VN, visual network; SMN, somatosensory network; DAN, dorsal attention network; VAN, ventral attention network; LN, limbic network; FPN, frontoparietal network; DMN, default mode network; L, left; R, right | | | |

| **Supplementary TABLE 2** The specific pairs of intrinsic functional connectivity showing significant differences between FCon and HC groups using network-based statistical analysis. | | | | | | | | |
| --- | --- | --- | --- | --- | --- | --- | --- | --- |
| **Seed region** | **fsaverage5 Coordinates** | | | | **Target region** | **fsaverage5**  **Coordinates** | | |
|  | **x** | | **y** | **z** |  | **x** | **y** | **z** |
| **VN-VN(3 connections)** | | | | | | | | |
| Vis9_L | -19 | | -89 | -7 | Vis18_L | -12 | -89 | 2 |
| Vis14_L | -38 | | -87 | -5 | Vis22_R | 38 | -76 | 12 |
| Vis29_L | -14 | | -87 | 26 | Vis25_R | 5 | -81 | 23 |
| **VN-SMN(4 connections)** | | | | | | | | |
| Vis30_L | -16 | | -82 | 32 | SomMot1_R | 53 | -3 | -6 |
| Vis23_R | 14 | | -91 | 14 | SomMot26_L | -35 | -22 | 59 |
| Vis3_R | 34 | | -56 | -15 | SomMot34_R | 20 | -32 | 62 |
| Vis23_R | 14 | | -91 | 14 | SomMot9_R | 41 | -12 | 21 |
| **VN-DAN(5 connections)** | | | | | | | | |
| Vis18_L | -12 | | -89 | 2 | DorsAttn-Post5_L | -27 | -67 | 24 |
| Vis22_L | -24 | | -70 | 6 | DorsAttn-Post14_L | -25 | -59 | 57 |
| Vis31_L | -21 | | -76 | 38 | DorsAttn-Post12_L | -35 | -51 | 52 |
| Vis31_L | -21 | | -76 | 38 | DorsAttn-PrCv2_L | -48 | -2 | 35 |
| Vis19_R | 15 | | -77 | 7 | DorsAttn-Post5_L | -27 | -67 | 24 |
| **VN-VAN(1 connection)** | | | | | | | | |
| Vis2_R | 37 | | -35 | -14 | SalVentAttn-TempOccPar4_R | 54 | -38 | 23 |
| **VN-LN(2 connections)** | | | | | | | | |
| Vis11_L | -10 | | -80 | -3 | Limbic-OFC3_R | 12 | 44 | -20 |
| Vis16_R | 44 | | -70 | 4 | Limbic-OFC1_R | 16 | 22 | -17 |
| **VN-FPN(4 connections)** | | | | | | | | |
| Vis9_L | -19 | | -89 | -7 | Cont-PFCl9_R | 47 | 26 | 29 |
| Vis13_L | -15 | | -43 | -5 | Cont-PFCl2_L | -43 | 32 | 9 |
| Vis20_R | 26 | | -66 | 5 | Cont-PFCl8_L | -36 | 30 | 34 |
| Vis17_R | 14 | | -48 | 3 | Cont-PFCmp1_R | 15 | 32 | 23 |
| **VN-DMN(9 connections)** | | | | | | | | |
| Vis3_L | -38 | | -60 | -18 | Default-pCunPCC4_L | -9 | -39 | 26 |
| Vis7_L | -18 | | -37 | -12 | Default-pCunPCC8_R | 14 | -53 | 36 |
| Vis8_L | -40 | | -70 | -7 | Default-PFC15_L | -24 | 46 | 30 |
| Vis14_L | -15 | | -43 | -5 | Default-pCunPCC2_L | -13 | -59 | 20 |
| Vis16_L | -18 | | -58 | -1 | Default-Temp4_R | 48 | -9 | -17 |
| Vis19_L | -22 | | -95 | 1 | Default-PFC15_L | -24 | 46 | 30 |
| Vis29_L | -14 | | -87 | 26 | Default-pCunPCC1_R | 12 | -55 | 16 |
| Vis19_R | 15 | | -77 | 7 | Default-Temp9_L | -62 | -33 | 3 |
| Vis7_R | 18 | | -37 | -10 | Default-PFCdPFCm12_R | 21 | 19 | 47 |
| **SMN-SMN(1 connection)** | | | | | | | | |
| SomMot11_L | -57 | | -18 | 22 | SomMot36_L | -19 | -40 | 66 |
| **SMN-DAN(1 connection)** | | | | | | | | |
| SomMot27_R | 32 | | -35 | 59 | DorsAttn-Post15_L | -12 | -56 | 60 |
| **SMN-VAN(1 connection)** | | | | | | | | |
| SomMot5_R | 34 | | -10 | 16 | SalVentAttn-Med4_L | -10 | 10 | 46 |
| **SMN-LN(1 connection)** | | | | | | | | |
| SomMot26_R | 37 | | -20 | 60 | Limbic-OFC1_L | -15 | 23 | -17 |
| **SMN-FPN(7 connections)** | | | | | | | | |
| SomMot1_L | -51 | | -12 | -1 | Cont-PFCl11_R | 34 | 29 | 34 |
| SomMot9_L | -59 | | -5 | 10 | Cont-Cing1_L | -6 | 3 | 30 |
| SomMot15_L | -52 | | -22 | 38 | Cont-PFCl12_R | 41 | 20 | 41 |
| SomMot26_L | -35 | | -22 | 59 | Cont-Par6_L | -46 | -46 | 41 |
| SomMot29_R | 32 | | -30 | 56 | Cont-PFCl5_L | -41 | 36 | 21 |
| SomMot40_R | 10 | | -35 | 73 | Cont-PFCv1_L | -29 | 16 | -7 |
| SomMot25_R | 40 | | -25 | 50 | Cont-pCun1_L | -12 | -75 | 43 |
| **SMN-DMN(6 connections)** | | | | | | | | |
| SomMot21_R | 49 | | -16 | 50 | Default-PFC11_L | -16 | 63 | 9 |
| SomMot6_R | 32 | | -24 | 14 | Default-PFC14_L | -14 | 52 | 29 |
| SomMot22_R | 40 | | -11 | 45 | Default-PFC21_L | -33 | 13 | 50 |
| SomMot7_R | 62 | | -28 | 6 | Default-pCunPCC9_R | 9 | -50 | 46 |
| SomMot8_R | 63 | | -36 | 11 | Default-pCunPCC9_R | 9 | -50 | 46 |
| SomMot19_R | 51 | | -20 | 41 | Default-PFCv1_R | 29 | 22 | -16 |
| **DAN-DAN(1 connection)** | | | | | | | | |
| DorsAttn-Post11_L | -31 | | -37 | 42 | DorsAttn-Post6_R | 53 | -29 | 41 |
| **DAN-VAN(1 connection)** | | | | | | | | |
| DorsAttn-Post9_R | 41 | | -30 | 38 | SalVentAttn-Med2_L | -10 | 1 | 39 |
| **DAN-LN(2 connections)** | | | | | | | | |
| DorsAttn-FEF3_L | -29 | | -9 | 45 | Limbic-OFC3_R | 12 | 44 | -20 |
| DorsAttn-Post16_R | 24 | | -57 | 58 | Limbic-OFC4_R | 19 | 41 | -16 |
| **DAN-FPN(1 connection)** | | | | | | | | |
| DorsAttn-Post11_R | 32 | | -48 | 39 | Cont-PFCl1_L | -40 | 46 | -8 |
| **DAN-DMN(5 connections)** | | | | | | | | |
| DorsAttn-FEF2_L | -26 | | 1 | 47 | Default-Temp5_R | 62 | -30 | -11 |
| DorsAttn-FEF4_L | -29 | | -9 | 45 | Default-Temp1_R | 54 | 5 | -32 |
| DorsAttn-PrCv2_L | -48 | | -2 | 35 | Default-pCunPCC9_L | -4 | -15 | 38 |
| DorsAttn-Post15_R | 8 | | -69 | 51 | Default-Temp5_L | -56 | 5 | -12 |
| DorsAttn-Post6_R | 53 | | -29 | 41 | Default-Par5_R | 41 | -65 | 40 |
| **VAN-VAN(1 connection)** | | | | | | | | |
| SalVentAttn-PFCl1_R | 31 | | 36 | 23 | SalVentAttn-Med7_R | 8 | -3 | 64 |
| **VAN-LN(1 connection)** | | | | | | | | |
| SalVentAttn-Med1_L | -12 | | 20 | 30 | Limbic-OFC3_R | 12 | 44 | -20 |
| **VAN-FPN(2 connections)** | | | | | | | | |
| SalVentAttn-PFCl1_R | 31 | | 36 | 23 | Cont-Par3_L | -49 | -53 | 38 |
| SalVentAttn-Med8_R | 15 | | 4 | 63 | Cont-PFCv1_R | 28 | 23 | -5 |
| **VAN-DMN(3 connections)** | | | | | | | | |
| SalVentAttn-TempOccPar4_R | | 54 | -38 | 23 | Default-Par7_L | -48 | -64 | 40 |
| SalVentAttn-Med4_R | 9 | | 10 | 56 | Default-pCunPCC2_L | -13 | -59 | 20 |
| SalVentAttn-Med1_R | 13 | | 16 | 34 | Default-pCunPCC7_R | 4 | -19 | 38 |
| **LN-LN(3 connections)** | | | | | | | | |
| Limbic-TempPole1_L | -38 | | -6 | -43 | Limbic-TempPole1_R | 32 | -2 | -36 |
| Limbic-TempPole2_L | -26 | | 5 | -41 | Limbic-TempPole3_R | 39 | 11 | -38 |
| Limbic-TempPole3_L | -27 | | -11 | -33 | Limbic-TempPole1_R | 32 | -2 | -36 |
| **LN-FPN(1 connection)** |  | | | | | | | |
| Limbic-TempPole2_L | -38 | | -6 | -43 | Cont-PFCl8_R | 23 | 50 | 22 |
| **LN-DMN(2 connections)** | | | | | | | | |
| Limbic-OFC4_L | -4 | | 21 | -22 | Default-pCunPCC6_R | 10 | -39 | 36 |
| Limbic-TempPole5_R | 33 | | 9 | -33 | Default-PFCdPFCm3_R | 9 | 41 | 3 |
| **FPN-DMN(1 connection)** |  | | | | | | | |
| Cont-pCun2_R | 9 | | -63 | 44 | Default-Par6_L | -37 | -74 | 39 |
| **DMN-DMN(3 connections)** | | | | | | | | |
| Default-PFC6_L | -29 | | 51 | -2 | Default-PFC14_L | -14 | 52 | 29 |
| Default-PFC21_L | -33 | | 13 | 50 | Default-Par5_R | 41 | -65 | 40 |
| Default-Temp7_R | 60 | | -43 | -3 | Default-pCunPCC1_R | 12 | -55 | 16 |
| The seed and target regions for the significant connections from this identified connectomic network were defined as belonging to the intrinsic functional brain networks based on the established Schaefer 400-area cortical parcellation template. | | | | | | | | |

| **Supplementary TABLE 3** Group differences in topological metrics between FCon and HC in regional level by using the Human Connectome Project Multi-Modal Parcellation(HCP-MMP) atlas. | | | | | | | | | |
| --- | --- | --- | --- | --- | --- | --- | --- | --- | --- |
| Metrics(AUC) | ROI | fsaverage5 coordinates | | | | | FCon(n=35)  Mean±SD | HC(n=40)  Mean±SD | *P* |
|  |  | x | y | | z | |  |  |  |
| Nodal efficiency | V3CD_R | 33 | | -84 | | 13 | 0.1585 ± 0.0026 | 0.1562 ± 0.0023 | 0.0001 |
| FCon > HC |  |  |  |  |  |  |  |  |  |
| Abbreviations: AUC area under the curve; SD standard deviations. *P* < 0.05(FDR corrected). | | | | | | | | | |

| **Supplementary TABLE 4** Number and ratio of pair-wise FC difference between FCon and HC by using the HCP-MMP atlas. | | | | | | | | | | | | | | |
| --- | --- | --- | --- | --- | --- | --- | --- | --- | --- | --- | --- | --- | --- | --- |
| Networks | **VN** | | **SMN** | | **DAN** | | **VAN** | | **LN** | | **FPN** | | **DMN** | |
|  | Hypo | Hyper | Hypo | Hyper | Hypo | Hyper | Hypo | Hyper | Hypo | Hyper | Hypo | Hyper | Hypo | Hyper |
| **VN** | 1  (0.0754%) | 3  (0.2262%) |  | | | | | | | | | | | |
| **SMN** | 1  (0.0401%) | 1  (0.0401%) | 0  (0%) | 0  (0%) |  | | | | | | | | | |
| **DAN** | 2  (0.0874%) | 1  (0.0437%) | 0  (0%) | 1  (0.0473%) | 1  (0.1057%) | 2  (0.2114%) |  | | | | | | | |
| **VAN** | 1  (0.0418%) | 0  (0%) | 0  (0%) | 0  (0%) | 1  (0.0494%) | 1  (0.0494%) | 0  (0%) | 0  (0%) |  | | | | | |
| **LN** | 1  (0.0641%) | 0  (0%) | 1  (0.0694%) | 2  (0.1389%) | 0  (0%) | 0  (0%) | 1  (0.0725%) | 0  (0%) | 2  (0.4598%) | 0  (0%) |  | | | |
| **FPN** | 1  (0.0401%) | 2  (0.0801%) | 0  (0%) | 0  (0%) | 2  (0.0947%) | 3  (0.1420%) | 2  (0.0906%) | 2  (0.0906%) | 1  (0.0694%) | 0  (0%) | 3  (0.2660%) | 0  (0%) |  | |
| **DMN** | 3  (0.0641%) | 3  (0.0641%) | 2  (0.0463%) | 1  (0.0231%) | 0  (0%) | 2  (0.0505%) | 1  (0.0242%) | 2  (0.0483%) | 0  (0%) | 1  (0.0370%) | 1  (0.0231%) | 2  (0.0463%) | 1  (0.0250%) | 2  (0.0499%) |
| Values in the first line of each cell are the count absolute number of suprathreshold edges associated with every pair of networks within the significant cluster acquired from the NBS analysis. In contrast, values in the second line indicate the percentage ratio of these numbers to the total number of connections for each network pair. Hypo cells represent hypoconnectivity (FCon < HC), whereas hyper cells represent hyperconnectivity (FCon > HC). | | | | | | | | | | | | | | |


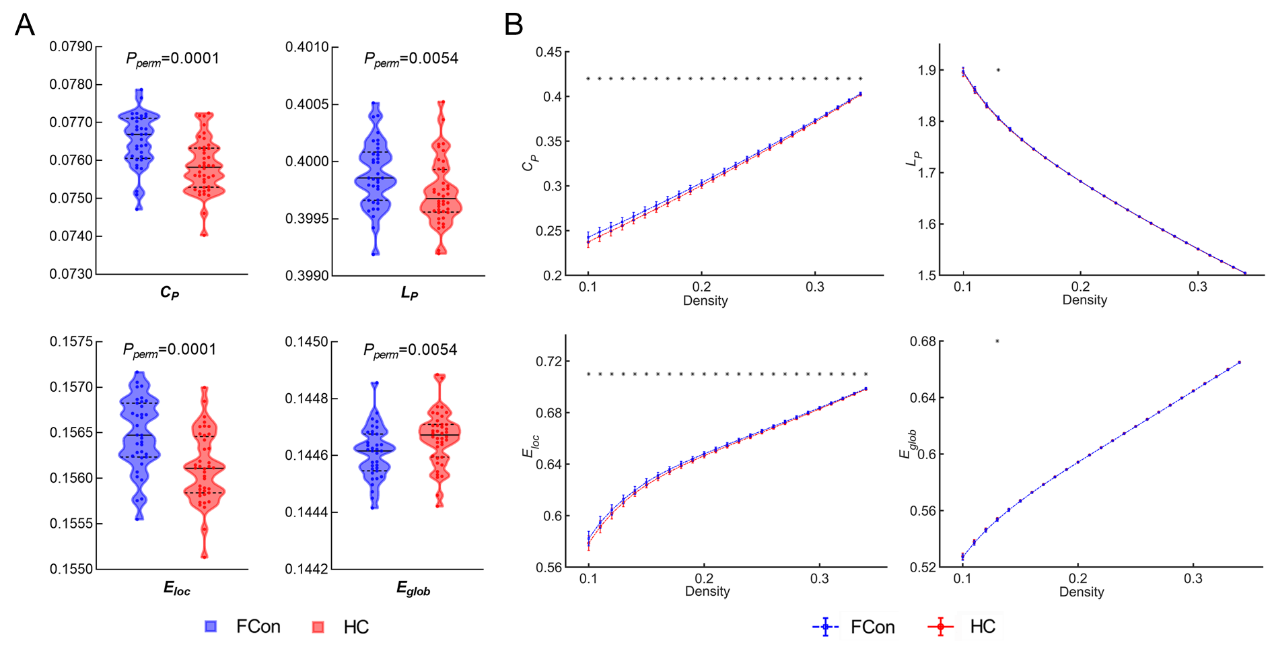


**Supplementary FIGURE 1** Between-group differences in global network topological metrics by using the HCP-MMP atlas.(A) Violin plots show the area under the curve (AUC) parameters of the clustering coefficient (C*_P_*), characteristic shortest path length (L*_P_*), local efficiency (E*_loc_*) and global efficiency (E*_glob_*) for patients with FCon and HC. (B) C*_P_*, E*_loc_*, L*_P_* and E*_glob_* across a wide range of density thresholds between 10 and 34%. Each point and error bar denote the mean and standard deviation at each density level, respectively. * indicates a significant difference at a given density threshold. (*P*<0.05, FDR correction).


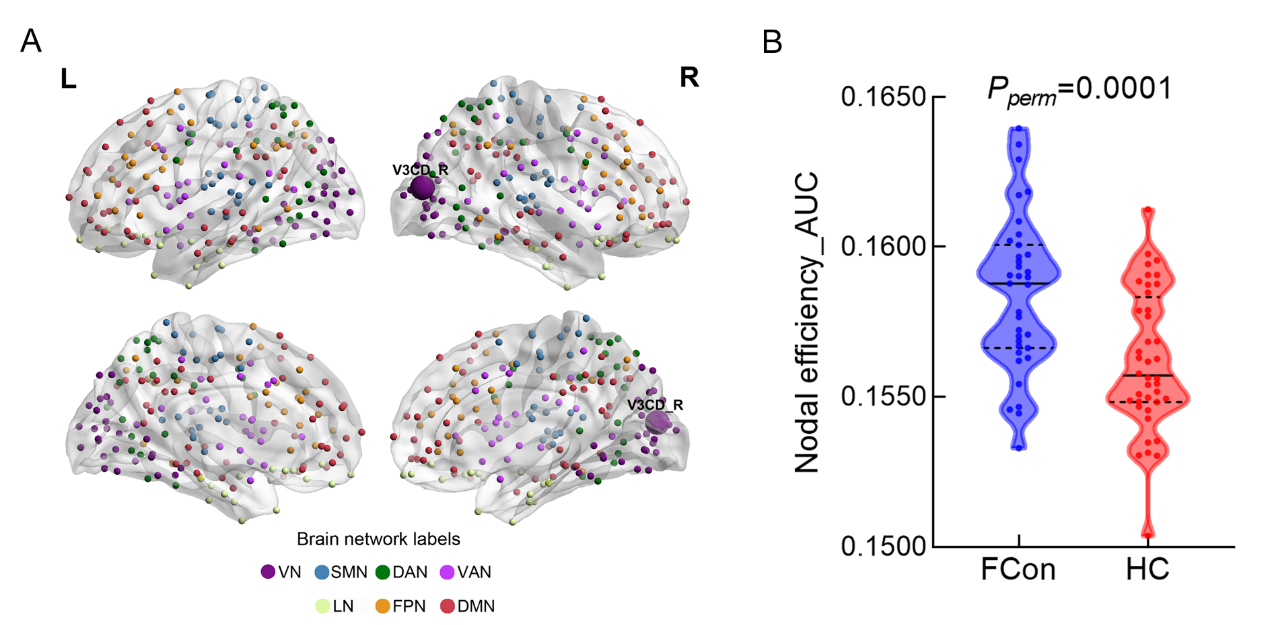


**Supplementary FIGURE 2** Between-group difference in nodal efficiency at regional level by using the HCP-MMP atlas. (A)There was significant difference in the V3CD_R which reported based on Yeo 7 networks. (B) Violin plot shows the AUC metrics of the brain regions with significant difference in nodal efficiency for patients with FCon and HC. (*P*<0.05 after FDR correction). For network abbreviation, please refer to the legend of Figure 2.


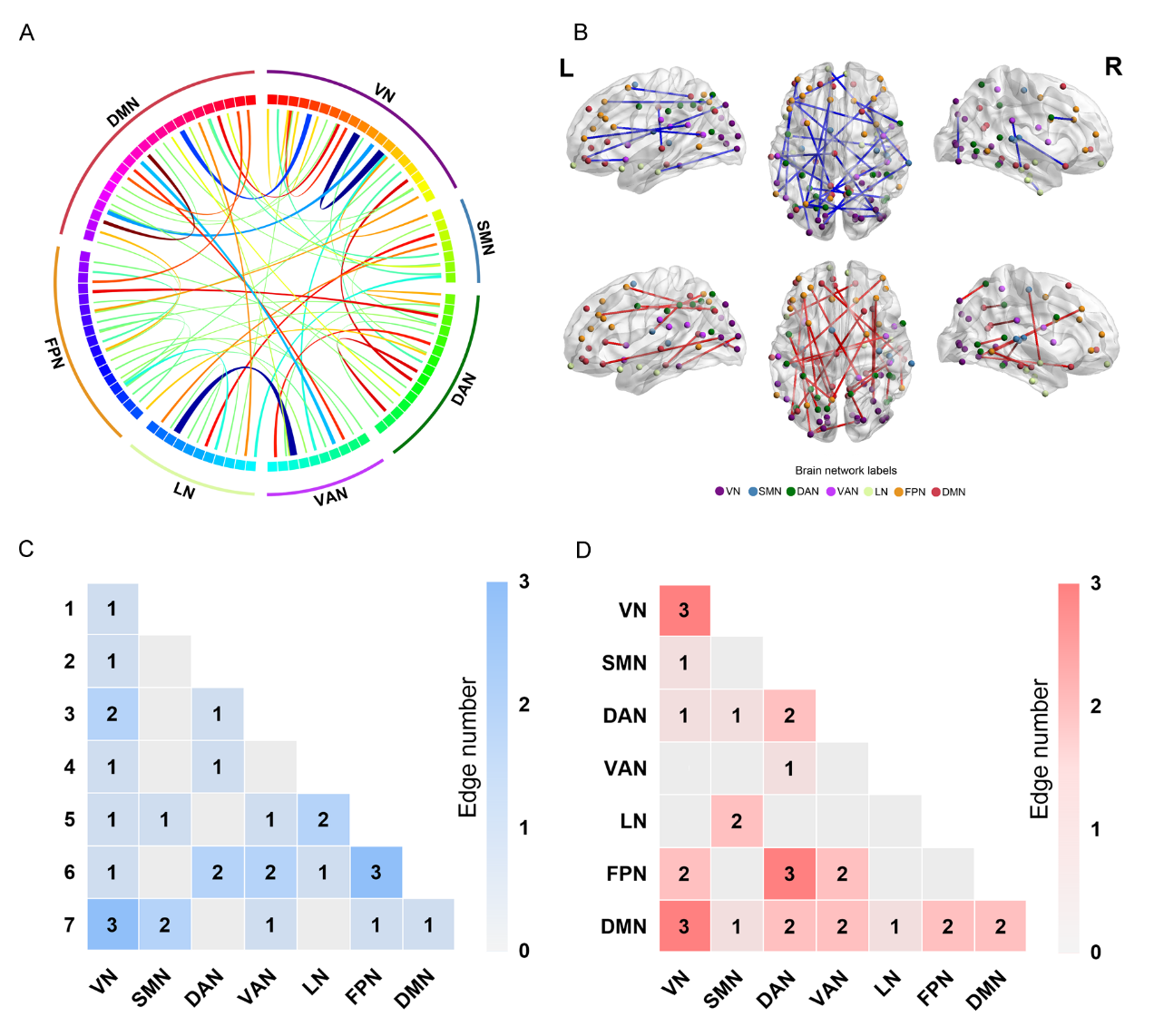


**Supplementary FIGURE 3** Differences in FC between FCon and HC in sub-network analysis. (A,B) The 101 nodes defined by the HCP-MMP atlas together with significant 60 unique yeo 7 network-pair edges in patients with FCon are list in circos and brain map, respectively.(C,D)Heatmaps show the number of significant edges for each pair of networks in group differences. Blue indicates that the FC is decreased while red indicates that the FC is increased compared with HC. For the color of edges, warm color indicates the FC is increased while cool color indicates the FC is decreased.(*P*<0.001 after NBS correction). For network abbreviation, please refer to the legend of Figure 2.


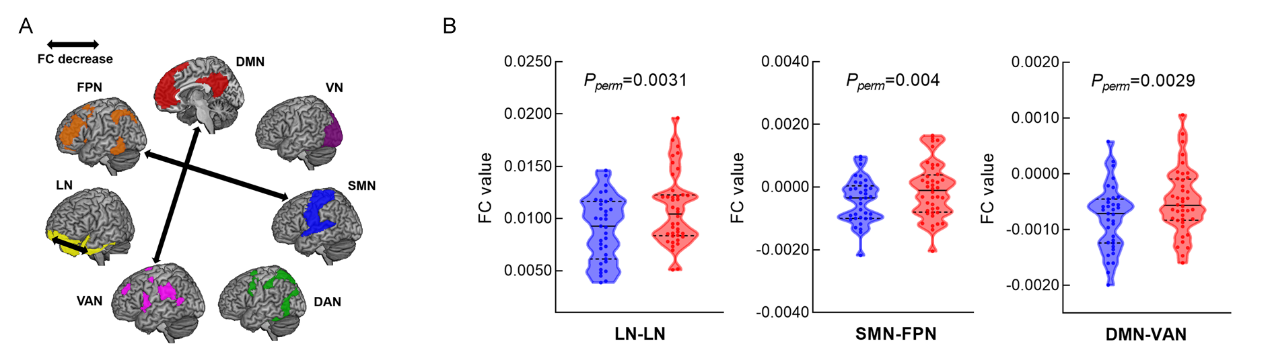


**Supplementary FIGURE 4** Differences in large-scale network between FCon and HC. (A) The schematic diagram shows the network connections with significant FC decrease within LN-LN and between-networks in the SMN-FPN and DMN-VAN. (B) Violin plots show FC value of the significant difference within- and between-networks for FCon and HC, respectively. (FDR-corrected *P* < 0.05). For network abbreviation, please refer to the legend of Figure 2.
